# Impacts of sunspot number and Geomagnetic aa-index on climate of Wet Zone West Africa during solar cycles 22–24

**DOI:** 10.1038/s41598-021-90999-6

**Published:** 2021-06-01

**Authors:** Esther A. Hanson, Francisca N. Okeke

**Affiliations:** 1 Department of Physics and Astronomy, University of Nigeria, Nsukka, Nigeria; 2 Advanced Space Technology Applications Laboratory, University of Uyo Main Campus, Uyo, Akwa Ibom State Nigeria; 3 NASA Goddard Space Flight Center, Greenbelt, MD USA

**Keywords:** Climate sciences, Astronomy and planetary science

## Abstract

Using the facilities at Heliophysics Science Division of NASA Goddard Space Flight Center, Greenbelt, MD, USA, we attempted to investigate the impact of solar magnetic activities on the climate of Wet Zone West Africa. The solar activity data namely, Sunspot Number (SSN) was obtained from the Royal Observatory of Belgium, Brussels; and Geomagnetic aa-index was obtained from World Data Center, Kyoto, Japan. Surface Air Temperature (SAT) and Rainfall data [for Port Harcourt in Nigeria and Abidjan in Cote D’Ivoire] were obtained from the HadCRUT-4 project of Climate Research Unit of University of East Anglia, United Kingdom. Firstly, we carried out Time Series Analysis of SSN and Geomagnetic aa-index spanning from 1950 to 2016. Secondly, we performed Regression Analysis on both solar activity data and climate variables to estimate the impact of solar magnetic activity on the Wet Zone West African climate. The Time Series Analysis showed that SSN variation was in-phase with Geomagnetic aa-index in all the solar cycles studied. Thus, Geomagnetic aa-index can be used as a proxy for studying solar magnetic activities. Performance of Regression Analysis showed that SSN regressed on SAT and Rainfall amounted to an average of 0.49 and 0.02% respectively throughout Solar Cycles 22–24. Furthermore, a regression of Geomagnetic aa-index on SAT and Rainfall yielded an average of 0.145 and 0.125% respectively. Our models showed that the variability of SAT and Rainfall in Wet Zone West Africa during Solar Cycles 22–24 are far less than 1%. Hence, the influence of SSN and Geomagnetic aa-index on SAT and Rainfall is less than 1%; and could cause ‘very small’ effect. These weak impacts are proofs that the variability of SAT and Rainfall were most probably not effected by SSN and Geomagnetic aa-index. Consequently, the variability of SAT and Rainfall in Wet Zone West Africa could not be attributed to SSN and Geomagnetic aa-index. We therefore, attempt to conclude that climate variability in Wet Zone West Africa is most probably not driven by solar magnetic activity, but could be attributed to anthropogenic activities.

## Introduction

The study of solar activities coupling with climate is of immensely great importance to both industries and academics due to numerous reasons. First, radio waves propagation occurs in the earth’s ionosphere, where radio communications deploying mobile phones, radar systems, shortwave broadcasters for radio stations, navigation systems for ships and avionics for aircrafts are made possible. Also, it is important to study climate change to curb its devastating effects on human, animal, aquatic lives and the environment. Infrastructural decay caused by flood and La Nina are unquantifiable. Adverse weather events such as thunder storm can trip-off electricity power lines and transponders on communications masts; creating huge financial losses to industries. During geomagnetic storms high electric currents flowing into the earth’s lower atmospheres and on the ground can cascade voltages resulting in power outages. High density of cosmic ray incidence causes damages to electrical components in aircrafts and space-crafts. Furthermore, heat waves result in health conditions for humans. Thus, by studying climate, preservation of human life and safety of property is attained. Nonetheless, best practices in Agriculture and Food Security have a strong forcing with research in climate change.

The influence of Galactic Cosmic Rays (GCRs) on cloud formation is suggested to be a veritable mechanism of solar activity influence on climate. The physical mechanism controlling the coupling of solar magnetic activity and climate is the incidence of GCRs which act as nucleation particles for cloud formation. Recently, an evidence of contribution of GCR to climate was presented in a scientific study^1^.

Historically a statistical study of GCR intensity and geomagnetic aa-index carried out^2^, and it showed a strong negative correlation, with a correlation coefficient of approximately − 0.86. Also, geomagnetic aa-index showed high correlation with solar activity data^3^. It is well established that GCR is a proxy index for solar activity. Used as proxies for solar activity^4–6^, these research showed that GCR variations are constantly out-of-phase with SSN.

Climate research is of huge significance because there are far-reaching, adverse effects of climate perturbations on human lives and sustainability development. Changing climate patterns have crippled economic activities on a global scale^7–9^. These are pointed to be driven by anthropogenic mechanisms such as industrialization, greenhouse gas emission and fossil fuel burning. It has been reported^10,11^ that the afore-mentioned processes result ultimately in increased world-wide temperatures^12^, causing melting of glaciers in North Atlantic and Pacific Oceans. Other consequences of climate change are hurricanes and tornadoes in the American sector; heat waves in the Asian sector; La Nina and El Nino in the Indian region. In the West African Sub-region, changing climate patterns have resulted in floods with associated negative impacts. Apart from the human-induced drivers of climate change, there has been hot debates among scientists^6,13–16^ as to whether solar magnetic activities contribute to climate change or not. Though their findings were published for other sectors of the world such as South Africa, India, America, South America, Europe^10,14,17–19^ from the available literature, researches on climate carried out in the West African Sub-region do not have solar activities content. Hence, in this research we analyzed solar activities proxy namely Sunspot Number (SSN) spanning from 1950 to 2016; and geomagnetic aa-index covering 1964–2016 to observe their associations with climate parameters in the Gulf of Guinea coastal belt of North Atlantic Ocean referred to as the Wet Zone West Africa.

In the West African Sub-region, changing climate patterns have resulted in floods with associated negative impacts such as destruction of public infrastructures, forced displacement of humans from their homes, dwindling economies, extinction of wild lives, and destruction of aquatic animals. Apart from the human-induced drivers of climate change, there has been hot debates among scientists, as to whether solar magnetic activities contribute to climate change or not. Though their findings are published for other sectors of the world, unfortunately, researches on climate carried out in the West African Sub-region do not have solar activities content.

This research has previously been done in other geographic areas including America, parts of Europe, India and South Africa^11,16,20,21^, but in the Sub-Saharan Africa, notably in the West African Sub-region, this is a novel step in this area of study. Research on wet zone West Africa is quite limited as evidenced by scantiness of literature, especially in this area of study. A few works carried out in Nigeria and Cote D’Ivoire are predominantly on climate change impact on the countries, and not on the coupling of climate variables with solar magnetic activities. For instance, a review work focused on plausible adverse effects of climate change on livelihoods in Nigeria^22^. Again, another study was concerned with a general overview of climate impacts in Nigeria using mean annual and monthly temperature and rainfall^23^. Other works on climate of Nigeria^24–27^ are reviews, which variables are completely devoid of solar activities indices. However, a couple of researchers studied the effects of changing solar activity on climate, and observed minimal contribution of solar activities on climate^28^. This agrees with our study.

We therefore attempted to resolve the discrepancy surrounding the differing views on solar activities contribution to climate in the West African Sub-region. The main objective of this research was to study the influence of solar activities on Wet Zone West African climate. The specific objectives included to; (1) estimate the oscillations of SSN and Geomagnetic aa-index for the period under investigation; namely 1950–2016, (2) investigate the association of SSN with SAT and Rainfall during Solar Cycles 22–24, (3) estimate the association of Geomagnetic aa-index with SAT and Rainfall at the same period. Objectives (1) was aimed at utilization of SSN and Geomagnetic aa-index for establishing the 11-year Solar Cycle signatures for the period under investigation. It would be concluded that SSN and Geomagnetic aa-index amplitudes exhibit patterns of 11-year solar cycle for the three Solar Cycles studied. Subsequently, the results obtained validated the suitability of SSN and Geomagnetic aa-index as solar magnetic parameters with which SAT and Rainfall data were regressed to ascertain their associations as indicated in objectives (2) and (3).

This is the first time this research was carried out in the West African sub-region, notably the Wet Zone, which lies on the Atlantic Coast. Port Harcourt (Nigeria) and Abidjan (Cote D’Ivoire) were the specific areas of study; low-latitude cities with altitudes 16 and 50 m respectively as shown in Table 1. The data was analysed to observe the response of SAT and Rainfall to SSNr and Geomagnetic aa-index for Cycles 22–24. SAT and Rainfall form basic parameters characterizing the climate of a region. On the other hand, Sunspot Number and Geomagnetic aa-index are prime variables for measurement of magnetic activities of the Sun. The results attempted to quantify the contribution of solar magnetic activities to climate change. This has shed more light on the controversy surrounding the forcing of climate change. In this work, we made an inference as to whether climate change is exclusively caused by anthropogenic activities, or could have a component of solar magnetic forcing.

**Table 1:** Coordinates of climate data sources.

S/N	Station	Code	Country	Geor. Lat	Geor. Lon	Altitude (m)
1	Port Harcourt	PHC	Nigeria	4.8° N	7.8° E	16
2	Abidjan	ABD	Côte D’Ivoire	5.2° N	4.0° W	50

## Data source and theory

SSN data [1950–2016] was obtained from the Royal Observatory of Belgium, Brussels. SSN was calculated from Eq. (1);

1 $${R}_{SSN} =k (10g +s)$$

where *R* is the Sunspot Number also referred to as SSN; *k* is observatory scaling factor, *g* is the number of sunspot groups, and *s* is the number of individual sunspots.

Solar cycles have an average duration of approximately 11 years. Solar maximum and minimum refer to periods of maximum and minimum sunspot counts and a solar cycle spans from one sunspot minimum to the next. Solar cycles 22, 23 and 24 had durations of 9.9, 12.3 and 12 years respectively. However, our work concentrated on the entire periods of all the Solar Cycles studied.

Geomagnetic aa-index was obtained from World Data Center for Geomagnetism, Kyoto, Japan. The Geomagnetic *aa-*index is a simple global geomagnetic activity index. Geomagnetic aa-index is a 3-hourly index computed from the *K* indices from two approximately antipodal observatories and has units of 1 nT. Currently, the two observatories used are Hartland in the United Kingdom, and Canberra, Australia. Since it is based on data from only two observatories, it is the simplest of all the 3 hourly planetary indices. The main advantages of using *aa* indices are that the time series spans further back (to 1868) than any of the other planetary index time series; and also up-to-date values are produced and made available on a weekly basis.

SAT and Rainfall data [1950–2016] were obtained from the HADCRUT4 project of the Climate Research Institute of the University of East Anglia, United Kingdom. The climate data were generated from two stations in the Wet Zone West Africa namely Port Harcourt (latitude 4.8° N; longitude 7.8° E) in Nigeria, and Abidjan (latitude 5.2° N; longitude 4.0° W) in Cote D’Ivoire as shown in Table 1.

## Methods of data analysis

We employed the facilities at National Aeronautics and Space Administration Goddard Space Flight Center, (NASA-GSFC) Greenbelt, Maryland, USA and adopted Time Series Analysis and Regression Analysis on the solar magnetic, geomagnetic and climate datasets. Firstly, using Time Series Analysis technique, we analyzed SSN and Geomagnetic aa-index to observe and determine the largescale time-series trends of the variability of SSN and Geomagnetic aa-index. Secondly, we performed Regression Analysis of SSN to estimate its association with SAT and Rainfall for Solar Cycles 22–24. And thirdly, again, we performed Regression Analysis to ascertain the effect of Geomagnetic aa-index on SAT and Rainfall in the same study area for the Solar Cycles under investigation. Solar magnetic data—SSN and Geomagnetic aa-index—were the Independent Variables, while climate variables—SSN and Rainfall—were the Dependent Variables.

Computations of the monthly and annual mean SSN were obtained from the daily values using expressions in Eqs. (2) and (3) respectively;

2 $$M_{SSN} = \frac{1}{n}\mathop \sum \limits_{i = 1}^{n} D_{i}$$

3 $$A_{SSN} = \frac{1}{12}\mathop \sum \limits_{i = 1}^{n} M_{i}$$

where *M*_*SSN*_ is the monthly mean value while D_*i*_ is the daily value, *n* is the number of days, *A*_*SSN*_ is the annual mean value, *Mi* is the monthly value.

Time series of Geomagnetic aa-index were computed using Eq. (4);

4 $$D_{aa} = \frac{1}{8}\mathop \sum \limits_{i = 1}^{n} H_{i}$$

where D_*aa*_ is the daily mean value, H_*i*_ is the three hourly value.

5 $$M_{aa} = \frac{1}{8}\mathop \sum \limits_{i = 1}^{n} Daa_{i}$$

where M_*aa*_ is the daily mean value, D_aai_ is the daily mean value.

A Simple linear regression considers only one independent variable using the relation expressed in Eq. (6);

6 $$y={\beta }_{0}+{\beta }_{1}x+e$$

which is analogous to;

6a $$Rainfall={\beta }_{0}+{\beta }_{1}SSN+e$$

6b $$SAT={\beta }_{0}+{\beta }_{1}SSN+e$$

6c $$Rainfall={\beta }_{0}+{\beta }_{1}GCR+e$$

6d $$SAT={\beta }_{0}+{\beta }_{1}GCR+e$$

where β_0_ is the y-intercept, β_1_ is the slope (or regression coefficient) and *e* is the error term.

## Results and discussion

### Variations of SSN and geomagnetic aa-index

**Figure 1:**
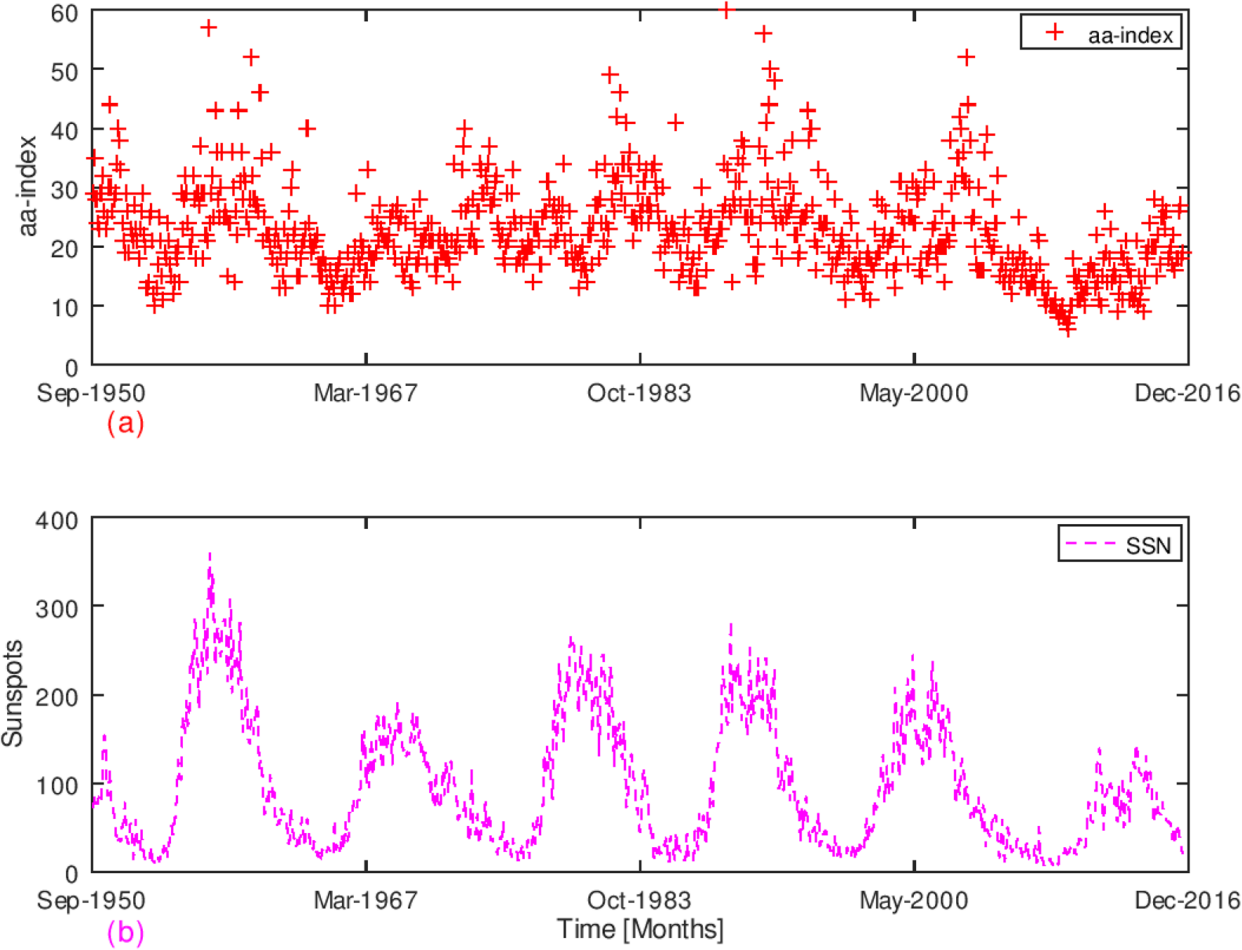
(**a**, **b**) Variations of Geomagnetic aa-index and SSN [September 1950 to December 2016]. The upper panel is the variation of geomagnetic aa-index obtained from the World Data Center of Geomagnetism, Kyoto, Japan spanning from solar cycle number 19 to solar cycle number 24. The lower panel [Magenta curve] is the variation of sunspots from solar cycle 19 to solar cycle 24. Periods of minimum solar magnetic activities correspond with periods of lowest magnitudes of geomagnetic aa-index. The Figure affirmed that sunspot variation was in-phase with geomagnetic aa-index. It was observed that solar cycle 19 was more active than preceding solar cycles, where SSN were 300 and 180 for solar cycles 19 and 20 respectively.

We analyzed the SSN and Geomagnetic aa-index of the period (1950–2016) which spans solar cycles 19, 20, 21, 22, 23 and 24 as shown in Fig. 1. The result showed that Geomagnetic aa-index [see panel (a) of Fig. 1] varies in-phase with SSN [see panel (b) of Fig. 1]; with highest and lowest values occurring at solar maxima and minima respectively. Solar Cycles 22, 23 and 24 had durations of 9.9, 12.3 and 12 years respectively. Solar cycle 22 had a maximum Sunspot Count of 212. And in Solar Cycle 23, Sunspots maximized at 180. Solar Cycle 24 had two peaks as predicted^29^; a maximum Sunspot Count of 99 in 2011 and another peak of 101 in 2014 as shown in Fig. 1b.

The Geomagnetic aa-index attained peak indices ranging between 35 and 58 in 1958, 1968, 1980, 1990, 2003 and 2014 which were solar maxima years. On the other hand, minimum values of aa-index ranging from 7 to 15 were recorded in 1953, 1965, 1976, 1987, 1997 and 2009 which were solar minima years. For instance, at the peak of solar maximum period in 1959, SSN attained a peak of 350 while Geomagnetic aa-index reached 60 nT. During solar cycle 24, the peak in SSN was approximately 110 and Geomagnetic aa-index value was at about 28 nT. Cycle 24 was shown to be the least active among the solar cycles studied. These results agreed with observations made by other researchers^30^. This implies that the geomagnetic field is directly driven by the Sun’s magnetic fields.

### Regression analyses of solar activity indices and climate variables

Regression Analysis is one of the reliable methods for estimation of association between variables; one considered independent while the other is the response or dependent variable. This method of analysis has proven to be a viable tool for attempting to address the research question ‘Whether solar magnetic activity could impact climate. The solar activity indices selected and subjected for the analyses were Sunspot Number (SSN) and Geomagnetic aa-index. On the other hand, the climate parameters chosen for this study were Surface Air Temperature and Rainfall. The datasets employed in this work spanned from Solar Cycle 22 towards the end of Solar Cycle 24, which started in September 1986 through December 2015 (see Table 2). We utilized such long-term dataset to attain a reliable, robust result in the course of estimating the impact of solar magnetic activity on climate of Wet Zone West Africa.

**Table 2:** List of solar cycles studied.

Solar cycle	Start date	Maximum	End date	Duration (year)
22	September 1986	November 1989	July 1996	9.9
23	August 1996	November 2001	November 2008	12.3
24	December 2008	April 2014	December 2019	11.0

In this study, SSN and Geomagnetic aa-index were the independent variables or Predictors, while SAT and Rainfall were the dependent or Response variables.

#### Regression of SSN on SAT and rainfall

**Figure 2:**
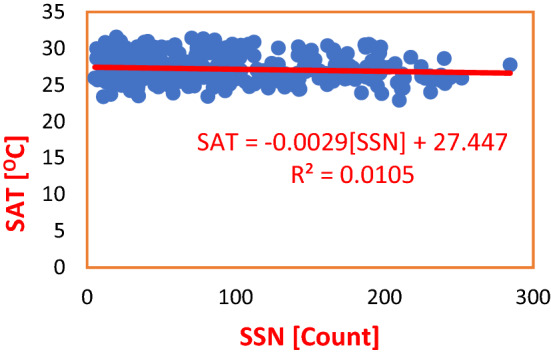
Regression of SSN on SAT in PHC during Solar Cycles 22–24. SSN made a 1% influence on SAT. The Regression Equation shows that a unit increase in SSN by 1 Count resulted to an insignificant decease in SAT by an average of 0.0029 ^O^C.

**Figure 3:**
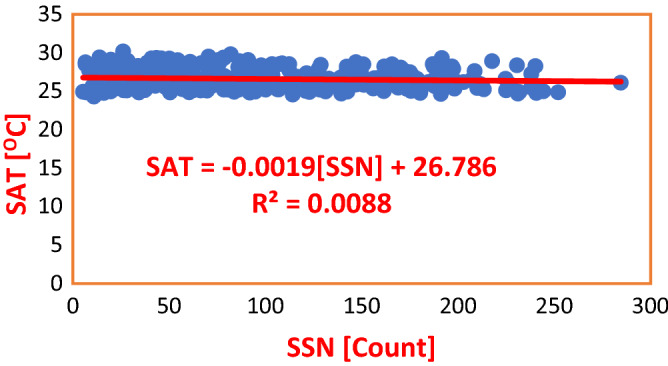
Regression of SSN on SAT in ABD during Solar Cycles 22–24. From the Regression Equation, SSN accounts for a negligible decrease in SAT by a mere 0.0019 °C. R^2^ value indicates that the impact of SSN on SAT is 0.8%, which is ‘too small’ to effect variation in SAT.

The association of solar magnetic activity and climate variables of the Wet Zone West Africa was estimated by performing a regression of SSN on SAT in Port Harcourt (Nigeria) and Abidjan (Cote D’Ivoire). The result showed that a unit increase in SSN count resulted to a 1% impact on SAT. From the Regression Equation, a unit count of SSN caused a decrease in SAT by 0.0029 °C as shown in Fig. 2. Also, regression of SSN on SAT in Abidjan showed that an increase in a unit count of SSN effected a decrease in SAT by 0.0019 °C, with Coefficient of Determination, ${R}^{2}=0.0088$, representing 0.8% impact of SSN on SAT as depicted in Fig. 3. The average impact of SSN on SAT was observed to be 0.49% as shown in Table 3.

**Table 3:** Regression equations for SSN regressed on SAT and rainfall in Nigeria and Cote D’Ivoire during solar cycles 22–24.

S/N	Climate station	Regression equation	Coefficient of determination	Influence (%)	Average influence (%)
	PHC	SAT = − 0.0029(SSN) + 27.447	R^2^ = 0.00105	0.10	0.49
	ABJ	SAT = − 0.0019(SSN) + 26.786	R^2^ = 0.0088	0.88	
	PHC	Rainfall = 0.0269(SSN) + 91.751	R^2^ = 0.0004	0.04	0.02
	ABJ	Rainfall = 0.002(SSN) + 112.01	R^2^ = 0.00003	0.003	

**Figure 4:**
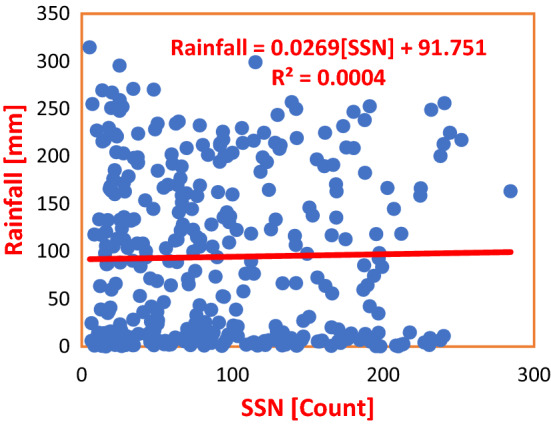
Regression of SSN on Rainfall in PHC during Solar Cycles 22–24. From the Regression Equation, SSN and Rainfall are not well associated. The Coefficient of Determination shows a 0.04% association, which is too insignificant to effect any change in the climate variable of the study area.

**Figure 5:**
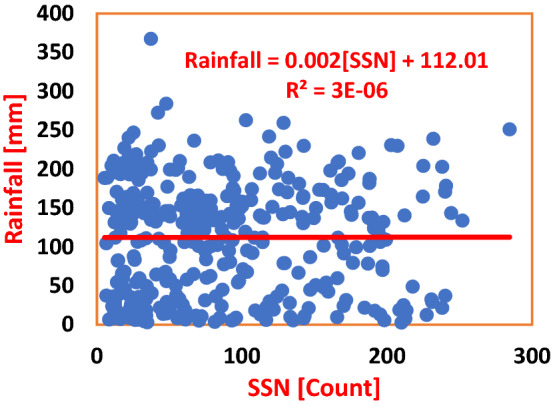
Regression of SSN on Rainfall in ABD during Solar Cycles 22–24. SSN made a 0.003% influence on SAT. The Regression Equation shows that a unit increase in SSN by I Count resulted to an insignificant decease in SAT by an average of 0.002 °C.

Figure 4 is the scatter plot of SSN and Rainfall showing the regression of the former on the latter. The Regression Equation shows that a unit count of SSN effected an increase in Rainfall by 0.0269 mm, with a corresponding Coefficient of Determination, ${R}^{2}=0.0004$. This value is too low to effect change in the climate of PHC within the period of Solar Cycles 22–24. Again, in Fig. 5, the Regression Equation shows that a unit count in SSN caused an increase in Rainfall by 0.002 mm in ABD. Here, SSN made a 0.003% impact on Rainfall. From the result, the impact is ‘very small’, and thus cannot effect a variation in the climate of the study area. Also, the average impact of SSN on Rainfall in both PHC and ABD amounted to 0.02% (see Table 3), which is too negligible for consideration as a parameter to forcing climate change in the Wet Zone West Africa.

#### Regression of geomagnetic aa-index on SAT and rainfall

**Figure 6:**
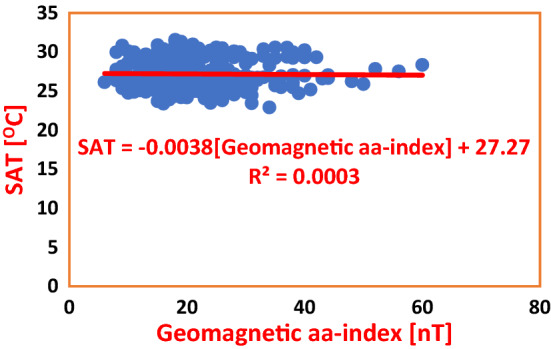
Regression of Geomagnetic aa-index on SAT in PHC during Solar Cycles 22–24. The Regression Equation indicates that Geomagnetic aa-index effected a decrease in SAT by 0.0038 °C. The Coefficient of Determination, R^2^ shows that the impact of Geomagnetic aa-index on variability of SAT is mere 0.03%.

**Figure 7:**
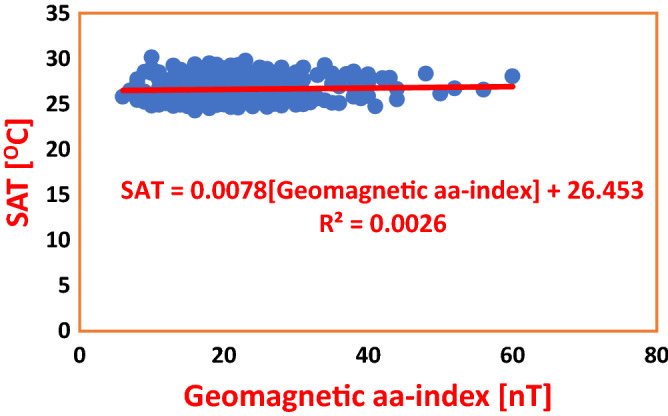
Regression of Geomagnetic aa-index on SAT in ABD during Solar Cycles 22–24. The model shows that an increase of Geomagnetic aa-index by 1 nT effected an increase in SAT by mere 0.0078 °C. Also, the Coefficient of Determination is too small to cause a tangible effect in variability of SAT.

Geomagnetic aa-index is one of the parameters for characterization of solar magnetic activity. We deployed it for statistical analysis to attempt estimation of the impact of solar activity on the climate of Wet Zone West Africa. A Regression Analysis technique was employed. The climate data utilized were SAT and Rainfall as earlier described in “Data source and theory” and “Methods of data analysis”. Figures 6 and 7 show the regression of Geomagnetic aa-index on SAT in PHC and ABD respectively, during Solar Cycles 22–24. The Coefficients of Determination yielded ${R}^{2}=0.0003$ and ${R}^{2}=0.0026$ respectively, resulting to 0.03 and 0.26% impacts of Geomagnetic aa-index on SAT in the two cities accordingly. The average impact of Geomagnetic aa-index on SAT was 0.145% as shown in Table 4.

**Table 4:** Regression equations for geomagnetic aa-index regressed on SAT and Rainfall in Nigeria and Cote D’Ivoire during solar cycles 22–24.

S/N	Climate station	Regression equation	Coefficient of determination	Influence (%)	Average influence (%)
	PHC	SAT = − 0.0038(aa-index) + 27.27	R^2^ = 0.0003	0.03	0.145
	ABJ	SAT = 0.0078(aa-index) + 26.453	R^2^ = 0.0026	0.26	
	PHC	Rainfall = − 0.347(aa-index) + 101.52	R^2^ = 0.0012	0.12	0.125
	ABJ	Rainfall = − 0.3073(aa-index) + 118.73	R^2^ = 0.0013	0.13	

**Figure 8:**
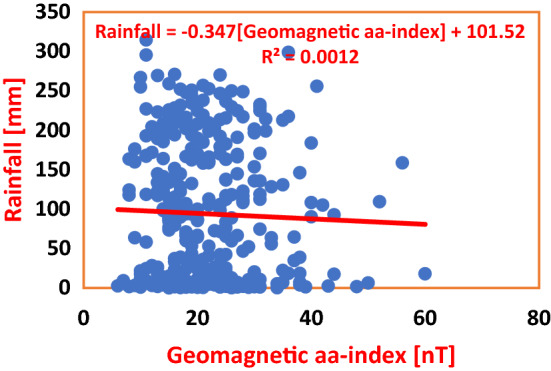
Regression of Geomagnetic aa-index on Rainfall in PHC during Solar Cycles 22–24. The percentage impact of Geomagnetic aa-index on Rainfall is observed to be 0.12%, which could only cause a ‘too small effect’. I surffices to state that the model shows that Rainfall variability in this study area is not forced by Geomagnetic aa-index.

**Figure 9:**
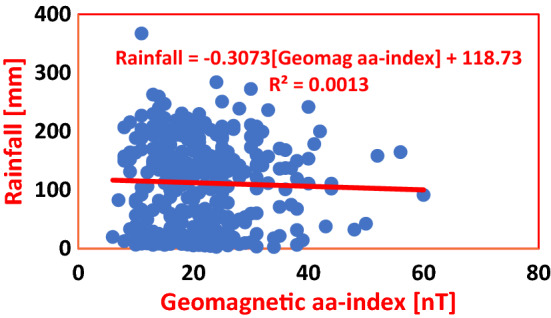
Regression of Geomagnetic aa-index on Rainfall in ABD during Solar Cycles 22–24. From the Regression Equation, a change in Geomagnetic aa-index by 1 nT caused a decrease in Rainfall by 0.3073 mm, with percentage variability of mere 0.13%. These values are too low for classification of Geomagnetic aa-index as a forcing on climate change in the study area.

Figure 8 depicts the association between Geomagnetic aa-index and Rainfall in PHC during the period under investigation. The Coefficient of Determination for regression of Geomagnetic aa-index on Rainfall yielded *R*^2^ = 0.0012, where a unit increase in Geomagnetic aa-index by 1 nT caused a decline in Rainfall by 0.347 mm. And the percentage impact of Geomagnetic aa-index on Rainfall was 0.12%. Figure 9 shows the Regression of Geomagnetic aa-index on Rainfall in ABD, where a unit increase in Geomagnetic by 1 nT effected a decrease in Rainfall by 0.3075 mm in ABD. The Coefficient of Determination *R*^2^ = 0.0013 is indicative of a 0.13% impact of Geomagnetic aa-index on Rainfall. The overall, average percentage impact of Geomagnetic aa-index on Rainfall in the Wet Zone West Africa during Solar Cycles 22–24; putting into consideration the impact in PHC (see Fig. 8) and in ABD (see Fig. 9), amounts to 0.125% as shown in Table 4.

Regression Analysis models the association between a variable considered to be independent and the other considered as the dependent variable. In this study, the Independent Variables or Predictors are SSN and Geomagnetic aa-index, while the Dependent Variables or Responses are SAT and Rainfall. R^2^ is a statistical measure which represents the proportion of the variance of the Dependent Variables [SAT, Rainfall] that is effected by the Independent Variables [SSN, Geomagnetic aa-index] in the regression models. It is also called the Coefficient of Determination. R^2^ is estimated to be between 0 and 100%, where 0% means that the model does not explain the variability of the Dependent Parameter; and 100% indicates that the model explains all the variability of the Dependent Parameter. Statistically, ${R}^{2}=0.75$, ${R}^{2}=0.50$, ${R}^{2}=0.25$, and ${R}^{2}=0.02$, representing 75%, 50%, 25% and 2% variability of the Dependent Parameter and are termed ‘substantial’, ‘moderate’, ‘weak’ and ‘small’ effect sizes respectively.

From our study, it follows that on the average, 0.49% of the variation of SAT was caused by SSN, and 0.02% of variation of Rainfall could be attributed to SSN. On the other hand, our models estimate that on the average, 0.145% of variation of SAT was caused by Geomagnetic aa-index, while 0.125% of variation of Rainfall could be attributed to Geomagnetic-aa-index. These R^2^ values are statistically too low, falling far behind the margins of standards for acceptability of R^2^ values considered to explain the variability of Dependent Parameters. Our models show that the variability of SAT and Rainfall in Wet Zone West Africa during Solar Cycles 22–24 are far less than 1%. Consequently, the variability of SAT and Rainfall in Wet Zone West Africa cannot be attributed to SSN and Geomagnetic aa-index. Therefore, we attempt to conclude that climate change may most likely not be attributed to solar magnetic activity.

## Summary

Using the facilities at the Goddard Space Flight Center, Greenbelt, MD, USA, we attempted to investigate the influence of solar magnetic activities on the climate of Wet Zone West Africa. We carried out Time Series Analysis of the solar magnetic data; namely Sunspot Number and Geomagnetic aa-index. We also performed a Regression Analysis on both solar activity data and climate variables to estimate the impact of solar magnetic activity on the climate. The study area was Wet Zone West Africa; specifically, Port Harcourt in Nigeria and Abidjan in Cote D’Ivoire.

The Time Series Analysis has shown that Sunspot Number variation was in-phase with Geomagnetic aa-index in all the solar cycles studied—Solar Cycles 22–24. Thus, Geomagnetic aa-index can be used as a proxy for studying solar magnetic activities.

Performance of Regression Analysis showed that SSN regressed on SAT amounted to an average of 0.49% throughout Solar Cycles 22–24. And on average, the regression of SSN on Rainfall yielded a 0.02% as shown in Table 3. SSN exhibited very negligible, weak influence on SAT and Rainfall in the study area during the period under investigation. Furthermore, a regression of Geomagnetic aa-index on SAT and Rainfall yielded an average 0.145%, and 0.125% respectively as shown in Table 4. Our models show that the variability of SAT and Rainfall in Wet Zone West Africa during Solar Cycles 22–24 are far less than 1%. With *R*^2^ < 0.001, the influence of SSN and Geomagnetic aa-index on SAT and Rainfall is less than 1%; which could cause ‘very small’ effect. These weak percentage impacts prove that the variability of SAT and Rainfall were most probably not effected by SSN and Geomagnetic aa-index. Consequently, the variability of SAT and Rainfall in Wet Zone West Africa cannot be attributed to SSN and Geomagnetic aa-index.

We therefore, attempt to conclude that variability in climate parameters in the Wet Zone West Africa is most probably not caused by solar magnetic activity, but could be attributed to anthropogenic effects.

## Conclusions

Using the facilities at the Goddard Space Flight Center, Greenbelt, MD, USA, we attempted to investigate the impact of solar magnetic activities on Wet Zone West African climate. Time Series Analysis of datasets spanning 1950–2016 revealed that Sunspot Number and Geomagnetic aa-index variations are in-phase; exhibiting the 11-year periodicity of Solar Cycle signatures. We deduce that Geomagnetic aa-index can be used as a proxy for studying solar magnetic activities.

Performing a Regression Analysis of solar activity data and climate variables, we estimated the impact of solar magnetic activity on the climate of Wet Zone West Africa; specifically, Port Harcourt in Nigeria and Abidjan in Cote D’Ivoire. The study showed that SSN regressed on SAT and Rainfall amounted to an average of 0.49 and 0.02% respectively throughout Solar Cycles 22–24. Furthermore, a regression of Geomagnetic aa-index on SAT and Rainfall yielded an average 0.145%, and 0.125%, respectively. Our models revealed that the variability of SAT and Rainfall in Wet Zone West Africa during Solar Cycles 22–24 are far less than 1%. Hence, the impacts of SSN and Geomagnetic aa-index on SAT and Rainfall is less than 1%; and could only cause ‘very small’ effect. These weak percentage impacts prove that the variability of SAT and Rainfall were most probably not effected by SSN and Geomagnetic aa-index. Consequently, the variability of climate in Wet Zone West Africa cannot be attributed to SSN and Geomagnetic aa-index. We therefore, attempt to conclude that Sunspot Number and Geomagnetic aa-index do not influence climate variability in the Wet Zone West Africa. Since solar activity is not the main force driving the changes in the climate of the study area, probably climate variability could be driven by anthropogenic activities.

## References

[1] Sloan T (2013). Cosmic rays, solar activity and the climate. J. Phys. Conf. Series.

[2] Shea, M.A. & Smart, D.F. (1985). in *19th Cosmic Ray Conference* Vol. 4, 501–504.

[3] Lyatsky, W. & Khazanov, G.V. (2008). A new polar magnetic index of geomagetic activity and its application to monitoring ionospheric parameters. NASA technical report server (NTRS). AGU Conf. Proceedings.

[4] Marsh N, Svensmark H (2000). Cosmic rays, clouds, and climate. Space Sci. Rev..

[5] Kniveton DR, Todd MC (2001). On the relationship of cosmic ray flux and precipitation. Geophys. Res. Lett..

[6] Sloan T, Wolfendale A (2013). Cosmic rays, solar activity and climate. Environ. Res. Lett..

[7] Rayamajhee V, Guo W, Bohara AK (2020). The impact of climate change on rice production in Nepal. EconDisCliCha.

[8] Kalkuhl M, Weinz L (2020). The impact of climate conditions on economic production: Evidence from global panel of regions. J. Environ. Econ. Manag..

[9] Farzanegan MR, Feizi M, Gholipour HF (2020). Drought and property prices: Empirical evidence from provinces of Iran. EconDisCliCha.

[10] Zanchettin D, Rubino A, Traverso P, Tomasino M (2008). Impact of variations in solar activity on hydrological decadal patterns in Northern Italy. J. Geophys. Res..

[11] Gray LJ, Beer J, Geller M, Haigh JD, Lockwood M, Matthes K, Cubasch U, Fleitmann D, Harrison G, Hood L, Luterbacher J, Meehl GA, Shindell D, van Geel B, White W (2010). Solar influences on climate. Rev. Geophys..

[12] Tinsley B, Burns G, Zhou L (2007). The role of the global electric circuit in solar and internal forcing of clouds and climate. Adv. Space Res..

[13] Hoyt DV, Schatten KH (1997). The Role of the Sun in Climate Change.

[14] Chaudhuri S, Pal J, Guhathakurta S (2014). The influence of galactic cosmic ray on all India annual rainfall and temperature. Adv. Space Res..

[15] Tsuda T, Shepherd M, Gopalswamy N (2015). Advancing the understanding of the Sun-Earth interaction—The climate and weather of the Sun-Earth System (CAWSES) II program. Progress Earth Planet. Sci..

[16] Kancírová M, Kudela K, Erlykin AD, Wolfandale AW (2016). Relevance of long-term time-series of atmospheric parameters at a mountain observatory to models for climate change. J. Atmos. Sol-Terr. Phys..

[17] Mouël JL, Kossobokov V, Courtillot V (2009). A solar pattern in the longest temperature series from three stations in Europe. J. Atmos. Solar Terr. Phys..

[18] Pablo JDM, Buccino AP (2010). Long-term solar activity influences on South American rivers. J. Atmos. Solar Terr. Phys..

[19] Rampelotto PH, Rigozo NR, da Rosa MB, Prestes A, Frigo E, Souza Echer MP, Nordemann DJR (2012). Variability of rainfall and temperature (1912–2008) parameters measured from Santa Maria (29° 41′ S, 53° 48′ W) and their connections with ENSO and solar activity. J. Atmos. Solar Terr. Phys..

[20] Erlykin AD, Sloan T, Wolfendale AW (2009). Solar activity and the mean global temperature. Environ. Res. Lett..

[21] Haigh JD, Winning AR, Toumi R, Harder J (2010). An influence of solar spectral variations on radiative forcing of climate. Nature.

[22] Idowu AA, Ayoola SO, Opele AI, Ikenweivie NB (2011). Impact of climate change in Nigeria. J. Energy Environ..

[23] Akpodiogaga P, Odjugo O (2017). General overview of climate change impacts in Nigeria. J. Hum. Ecol..

[24] Olaniyi OA, Funmilaya OA, Olutinmehin IO (2010). Reviews of climate change and its effects on Nigeria ecosystem. Int. J. Environ. Pollut. Res..

[25] Enete, A.A. & Amusa, T.A. Challenges of agricultural adaptation to climate change in Nigeria: A synthesis from the literature. *J. Field Action Field Action Sci. Rep*. **4** (2010).

[26] Amobi, D. & Onyishi, T. Governance and climate change in Nigeria: A public policy perspective. *J. Policy Develop. Stud*. **9**(2). 10.12816/0011217 (2015).

[27] Akpomi, M. E. & Vipene, J. Promotion, knowledge of climate change amongst Nigerians: Implications for education managers. *J. Educ. Pract.* **7**(32). ISSN 2222-288X (2016).

[28] Engels, S. & Geel, B. The effects of changing solar activity on climate: Contributions from oalaeodimatological studies. *J. Space Weather Clim*. **2**. 10.1051/swsc/2012009 (2012).

[29] Pesnell WD (2015). Predictions of Solar Cycle 24: How are we doing?. Space Weather.

[30] Alexander WJR, Bailey F, Bredenkamp DB, Merwe A, Willemse N (2007). Linkages between solar activity, climate predictability and water resource development. J. S. Afr. Inst. Civil Eng..

